# Exploring Empirical Rank-Frequency Distributions Longitudinally through a Simple Stochastic Process

**DOI:** 10.1371/journal.pone.0094920

**Published:** 2014-04-22

**Authors:** Benjamin J. Finley, Kalevi Kilkki

**Affiliations:** Department of Communications and Networking, Aalto University, Espoo, Finland; Universita’ del Piemonte Orientale, Italy

## Abstract

The frequent appearance of empirical rank-frequency laws, such as Zipf’s law, in a wide range of domains reinforces the importance of understanding and modeling these laws and rank-frequency distributions in general. In this spirit, we utilize a simple stochastic cascade process to simulate several empirical rank-frequency distributions longitudinally. We focus especially on limiting the process’s complexity to increase accessibility for non-experts in mathematics. The process provides a good fit for many empirical distributions because the stochastic multiplicative nature of the process leads to an often observed concave rank-frequency distribution (on a log-log scale) and the finiteness of the cascade replicates real-world finite size effects. Furthermore, we show that repeated trials of the process can roughly simulate the longitudinal variation of empirical ranks. However, we find that the empirical variation is often less that the average simulated process variation, likely due to longitudinal dependencies in the empirical datasets. Finally, we discuss the process limitations and practical applications.

## Introduction

Rank-frequency laws, such as Zipf’s law and more generally power laws, have been observed in a multitude of domains ranging from the intensity of wars to the populations of major cities [1] [2] [3]. As more rank-frequency laws are observed empirically and as rank-frequency distributions are adopted by increasingly diverse fields such as economics and behavioral sciences the importance of practical modeling of rank-frequency distributions in general similarly grows.

Historically, empirical rank-frequency distributions became a focus of research as the ubiquity of the power law in many of these distributions became apparent. A famous example being Zipf’s observations relating word frequency to word rank in natural language texts [4]. However, often power law models do not accurately fit the final tail ranks of empirical rank-frequency distributions. Tail ranks are typically affected by, for example, real-world finite size effects [5]. Many researchers fit models up to a cutoff point or utilized combinations such as power law with an exponential tail. Recently, however, novel methodologies have been proposed. Ref. [6] for example, utilized a discrete version of the generalized beta distribution to accurately fit a large array of empirical rank-frequency distributions.

However, as far as we know these novel approaches have not examined the longitudinal variation of empirical rank-frequency distributions. Furthermore, many of these approaches understandably focus on rank-frequency distributions with power law like bodies (linear on a log-log scale). In contrast, we simulate several empirical rank-frequency distributions by using a simple stochastic cascade process (hereafter the fracturing (FT) process). The FT process is a stochastic multiplicative process that produces a concave rank-frequency distribution (on a log-log scale), due to an underlying relationship with the gamma distribution. In addition, the FT process cascades a fixed finite number of times and contains a minimum size constraint thus replicating finite size effects. Finally, we show that the FT process can roughly simulate the longitudinal variation of empirical ranks through repeated trials of the process. In other words, each FT process trial can represent an observation of the dataset at a specific point in time.

Importantly, we emphasize the practical application of the FT process rather than detailed mathematical derivations. Furthermore, we hope that the process’s simplicity will allow non-experts to understand the process and help illustrate how even a simple process can give rise to a variety of rank-frequency shapes.

## Results

**Figure 1:**
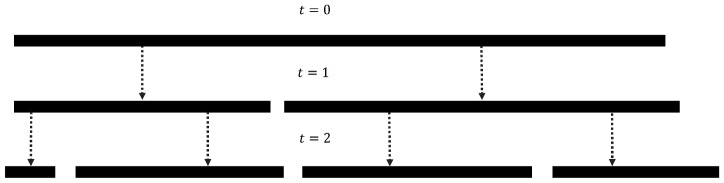
Visualization of FT process after two time steps. A visualization of the FT process after two time steps and thus four resultant intervals.

To start we give a brief overview of the FT process. Essentially the process simulates the repeated fracturing of an interval (or basically any one dimensional object). The process begins with a single unit interval at time step 0, and at every time step 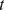 all existing intervals are fractured into exactly two smaller intervals. Figure 1 illustrates the process for two time steps. The fracturing point for each fracture is determined by a transformed standard uniform random variable. The transformation function contains a fitting parameter which is utilized to fit the FT process to the empirical distribution. After the final time step, all resultant intervals below a specific minimum size are set to the minimum. Finally, the intervals are sorted by length to create a rank-frequency distribution. In other words, the length of an interval is equivalent to the magnitude of a single rank. The FT process is described in greater detail in the Methods section.

The FT process is highly related to fracturing (also known as fragmentation) processes studied extensively by physicists and mathematicians [7] [8] [9]. Kolmogorov’s particle fracturing process is an early and prominent example [10]. Kolmogorov showed that under certain conditions the repeated fracturing of a particle (or interval) implies a log-normal size-frequency distribution through the central limit theorem. (Note the size-frequency and rank-frequency distributions are related [11].) Though, in contrast to Kolmogorov’s process, in the FT process all intervals are repeatedly fractured and the size-frequency distribution is related to the gamma distribution (see Analytical Form of FT Process section) [12]. More recently, a related stochastic cascade process that produces stretched exponential distributions was similarly presented as a complimentary alternative to the power law distribution [13]. A comprehensive theoretical overview of random fragmentation processes can be found in [14]. In summary, the FT process represents a classic fragmentation process that has been modified and reapplied.

### Process Fitting

We utilize the FT process to simulate six empirical datasets to illustrate the types of datasets well represented by the process. The empirical datasets focus mostly on consumer popularity and are as follows:

- French book sales volumes in 2003 [15]
- US theatrical earnings in 2002 [16]
- US last name frequency of census respondents in the 2000 census [17]
- Artist play frequency of Audioscrobbler music plugin as of May 2005 [18]
- YouTube video request frequency originating from the University of Massachusetts Amherst campus network during several dispersed observation periods in fall 2007 to spring 2008 [19]
- US magazine circulation revenue estimates in 2000 [20]

**Figure 2:**
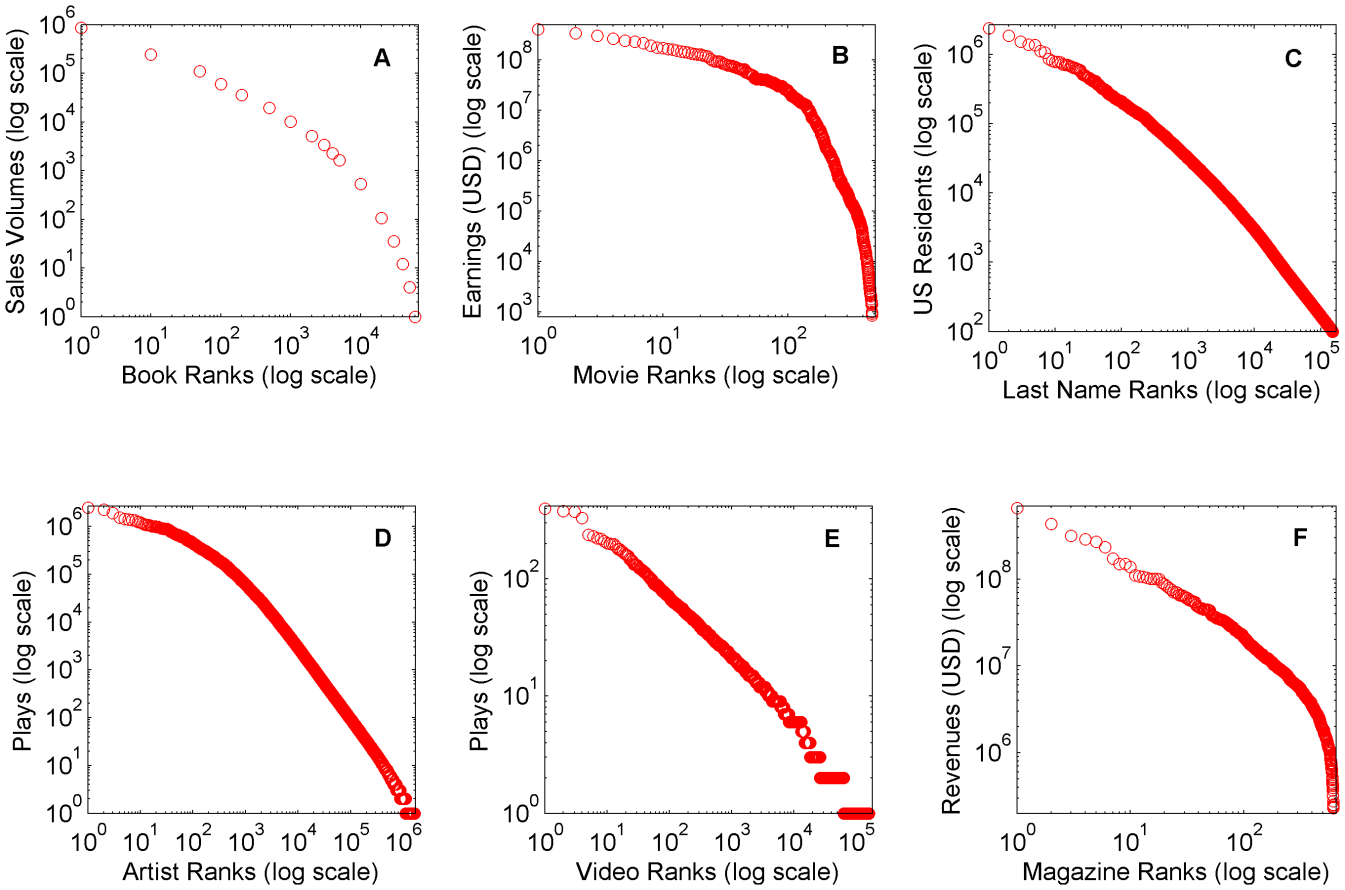
Rank-Frequency plots of empirical datasets. Rank-frequency plots of all empirical datasets utilized in the process fitting. These datasets are (A) French book sales volumes in 2003 [15], (B) US theatrical earnings in 2002 [16], (C) US last name frequency of census respondents in the 2000 census [17], (D) Artist play frequency of Audioscrobbler music plugin as of May 2005 [18], (E) YouTube video request frequency originating from the UMASS Amherst campus network during several dispersed observation periods in fall 2007 to spring 2008 [19], (F) US magazine circulation revenue estimates in 2000 [20].

Figure 2 illustrates the rank-frequency distributions for these datasets.

Furthermore, in order to establish the strengths of the FT process in relation to other popular models, we also fit a classical one parameter power law model and the previously mentioned novel discrete generalized beta distribution (DGBD). The power law and DGBD models were fit using multiple linear regression on the log-log transform of the models, in an approach similar to [21]. Methodological concerns about this type of log-log linear regression fitting have been expressed (Appendix A in [22]). However, since we aim to only briefly illustrate the types of datasets well represented by these models we set aside these concerns. More detailed fitting procedures for the power law and DGBD models are described in the Methods section.

Similarly, the log-log transform of the FT process was fit to the datasets by manually maximizing the coefficient of determination (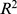). Specifically, we maximized 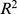 subject to manual variations of the FT process fitting parameter. (Note that in this case the maximization of 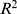 is equivalent to the typical minimization of the sum of square residuals). The fitting was performed manually because regression algorithms can become trapped in local minimums created by stochastic variations. In the manual fitting, the FT process results were averaged over 1000 trials to help eliminate some of these stochastic variations.

**Figure 3:**
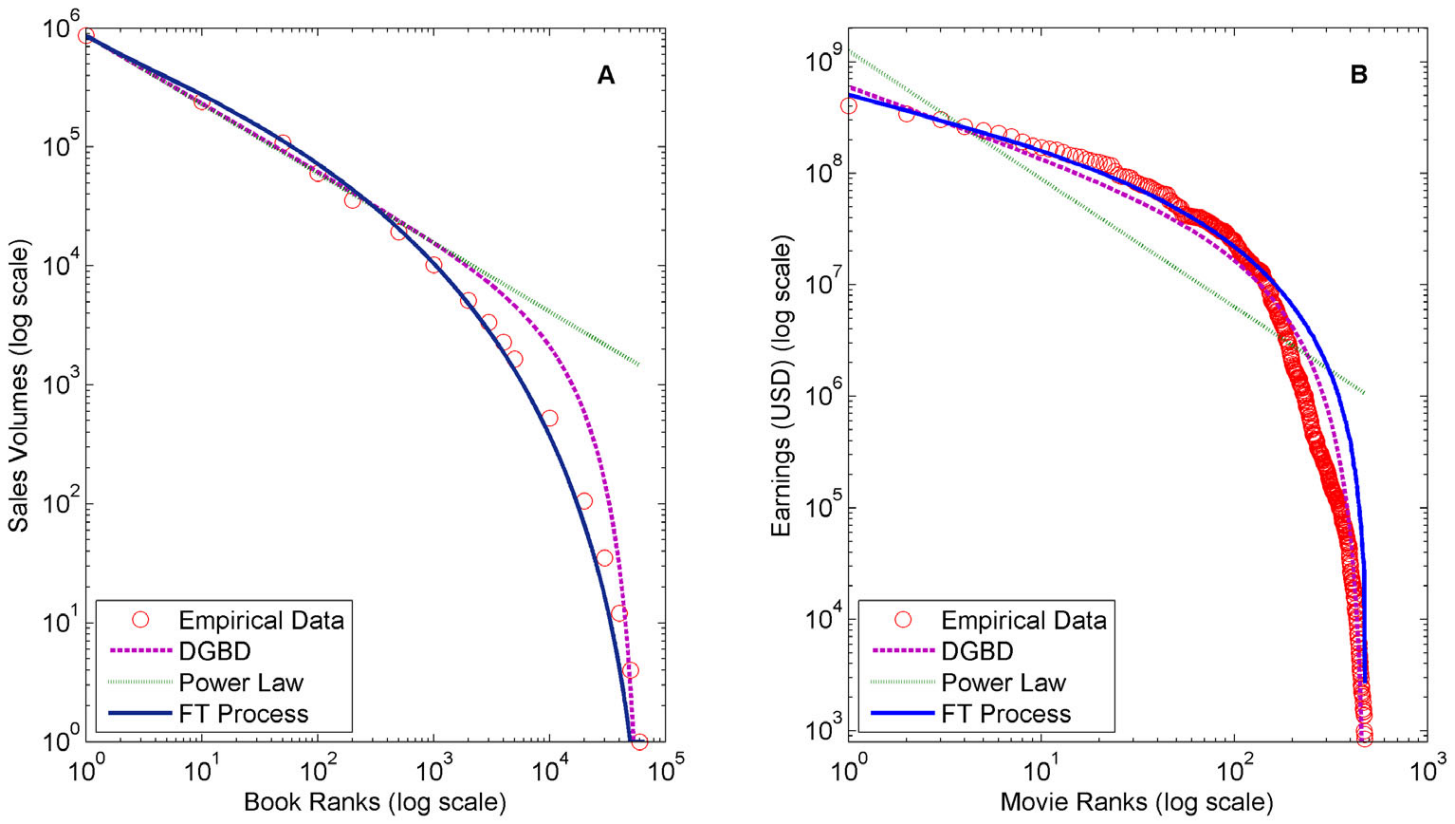
Comparison of fittings on (A) 2003 French book sales and (B) 2002 US theatrical earnings. A comparison of power law model, discrete generalized beta distribution, and FT Process fittings on (A) 2003 French book sales and (B) 2002 US theatrical earnings.

**Figure 4:**
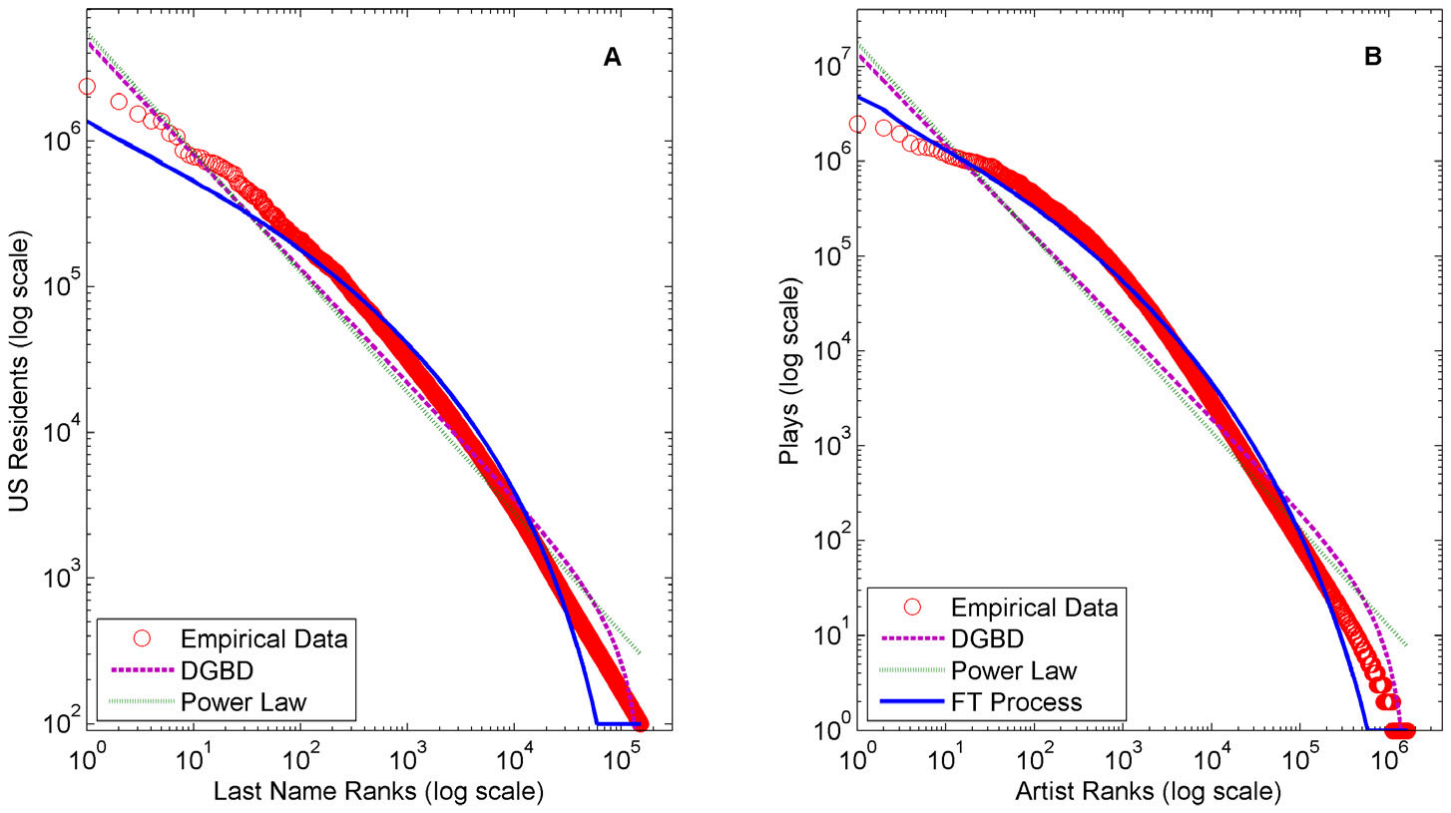
Comparison of fittings on (A) 2000 US Census last name frequency and (B) 2005 Audioscrobbler plugin artist plays. A comparison of power law model, discrete generalized beta distribution, and FT Process fittings on (A) 2000 US Census respondents last name frequency and (B) 2005 Audioscrobbler plugin artist plays.

**Figure 5:**
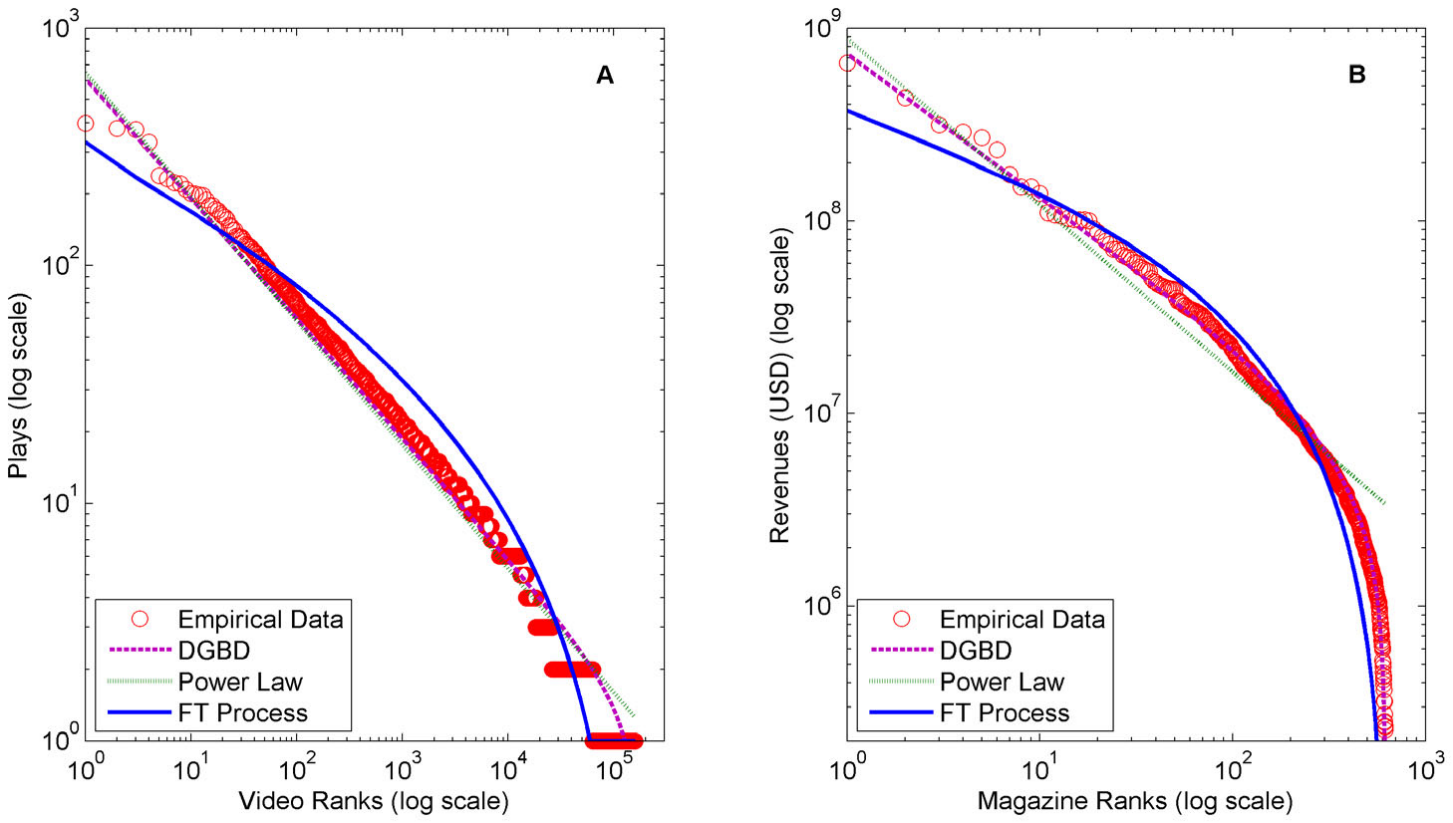
Comparison of fittings on (A) UMASS Amherst YouTube frequency and (B) 2000 US magazine circulation revenues estimates. A comparison of power law model, discrete generalized beta distribution, and FT Process fittings on (A) UMASS Amherst YouTube frequency and (B) 2000 US magazine circulation revenue estimates.

All models were fit using regression weights inversely proportional to their ranks. In other words, the weight of the first, second, and third ranked elements were 1/1, 1/2, and 1/3 respectively and so forth. This weighting scheme emphasizes highly ranked elements similar to logarithmic weighting [21]. We utilize this logarithmic fitting methodology because rank-frequency distributions are typically viewed on log-log scale and the emphasis of the head ranks is often useful in practice. Figures 3, 4, and 5 show the fittings of all datasets and Table 1 shows the 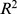 values for the different fittings. These 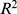 values take into account the weightings (the weighted 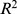 equation is detailed in the Methods section).

**Table 1:** Adjusted^1^ (and non-adjusted) coefficients of determination (R^2^) with weighting for fittings of empirical datasets.

Dataset	FT Process	Power Law	DGBD
French Book Sales (2003)	.9922 (.9927)	.9845 (.9855)	**.9933 (.9942)**
US Theatrical Earnings (2002)	**.9572 (.9572)**	.7146 (.7152)	.9541 (.9543)
Census Respondents Last Name Frequency	**.9849 (.9849)**	.9705 (.9705)	.9795 (.9795)
Audioscrobbler Music Artist Play Frequency	**.9883 (.9883)**	.9379 (.9379)	.9524 (.9524)
UMASS Amherst YouTube Video Request Frequency	.9748 (.9748)	.9823 (.9823)	**.9858 (.9858)**
US Magazine Circulation Revenue Estimates (2000)	.9212 (.9215)	.9633 (.9634)	**.9965 (.9965)**

1 The adjusted coefficient of determination is utilized because the FT process, Power Law, and DGBD have different numbers of regressors and the adjusted coefficient of determination utilizes a penalty for additional regressors that allows comparison.

Clearly the FT process and DGBD fit the truncated tails of several of distributions better than the classic power law model. The finite size effects of these datasets severely disrupt any kind of pure power law pattern.

The DGBD performs best on distributions with power law like bodies, such as the YouTube video request dataset (Figure 5A) and the US magazine circulation revenues dataset (Figure 5B). We define a power law like body as a power law relationship that spans several orders of magnitude. In fact, the power law model is a special case of the DGBD in which the tail also follows a power law (the analytic relationship between the models is shown in the Methods section). In contrast, the FT process performs best on distributions with concave bodies (on a log-log scale) such as the French book sales dataset (Figure 3A) and the Audioscrobbler artist plays dataset (Figure 4B). As discussed, the FT process is related to the gamma distribution, which is concave on a log-log scale.

Furthermore, the FT process inherently fits best rank-frequency distributions with reciprocal head and tail slopes, such as the French book dataset (Figure 3A) and US theatrical earnings dataset (Figure 3B). This property is the result of the common fracturing point distribution which is applied to every fracture irrespective of the interval value or process step.

### Longitudinal Variation

Next we simulate the longitudinal variation of several of the empirical datasets using the FT process. Our longitudinal datasets are expanded versions of three of the fitted datasets:

- French book sales volumes from 2003–2007 [15]
- US theatrical earnings from 2002–2012 [16]
- US magazine circulation revenue estimates from 2000–2012 [20]

**Figure 6:**
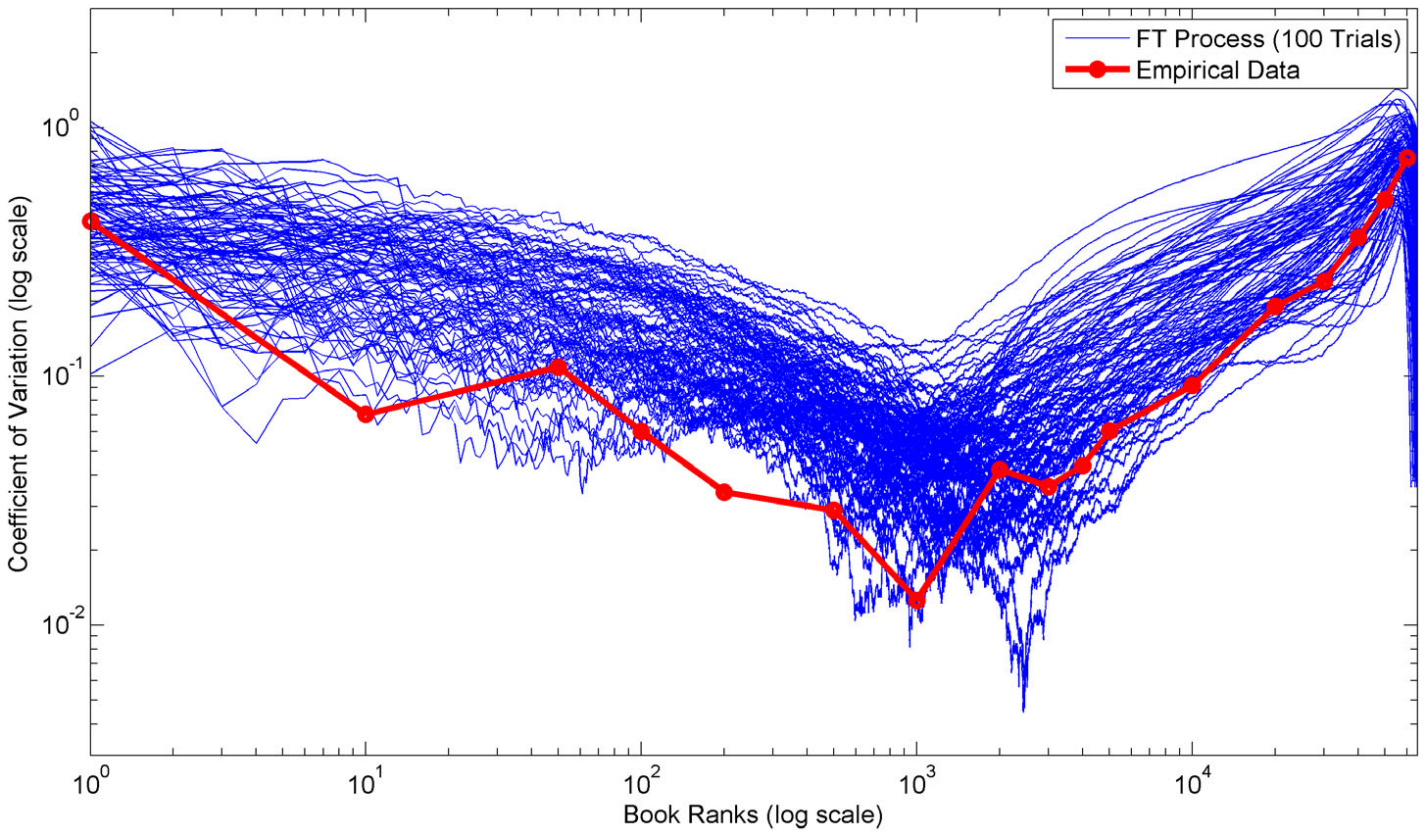
Empirical and simulated coefficients of variation for ranks of 2003–2007 French book sales. Plot of the coefficients of variation of ranks for the 2003–2007 French book sales dataset. The blue lines are simulated coefficients for 100 FT process trials, while the red line is the coefficients of the empirical data. Note that because the French book dataset consists of only 17 disparate ranks, the red points are empirical data points and the red lines are extrapolations.

**Figure 7:**
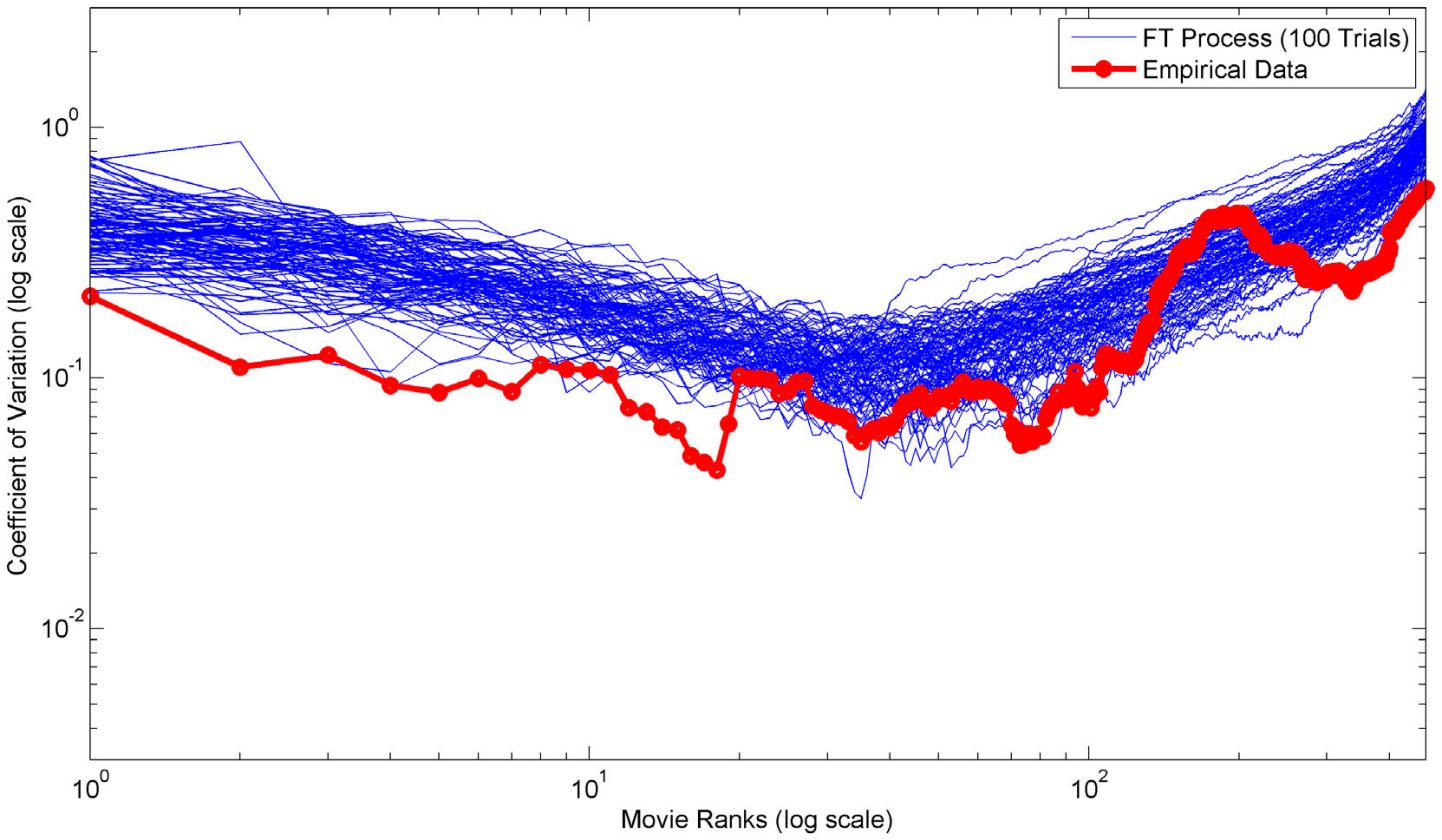
Empirical and simulated coefficients of variation for ranks of 2002–2012 US theatrical earnings. Plot of the coefficients of variation of ranks for the 2002–2012 US theatrical earnings dataset. The blue lines are simulated coefficients for 100 FT process trials, while the red line is the coefficients of the empirical data.

**Figure 8:**
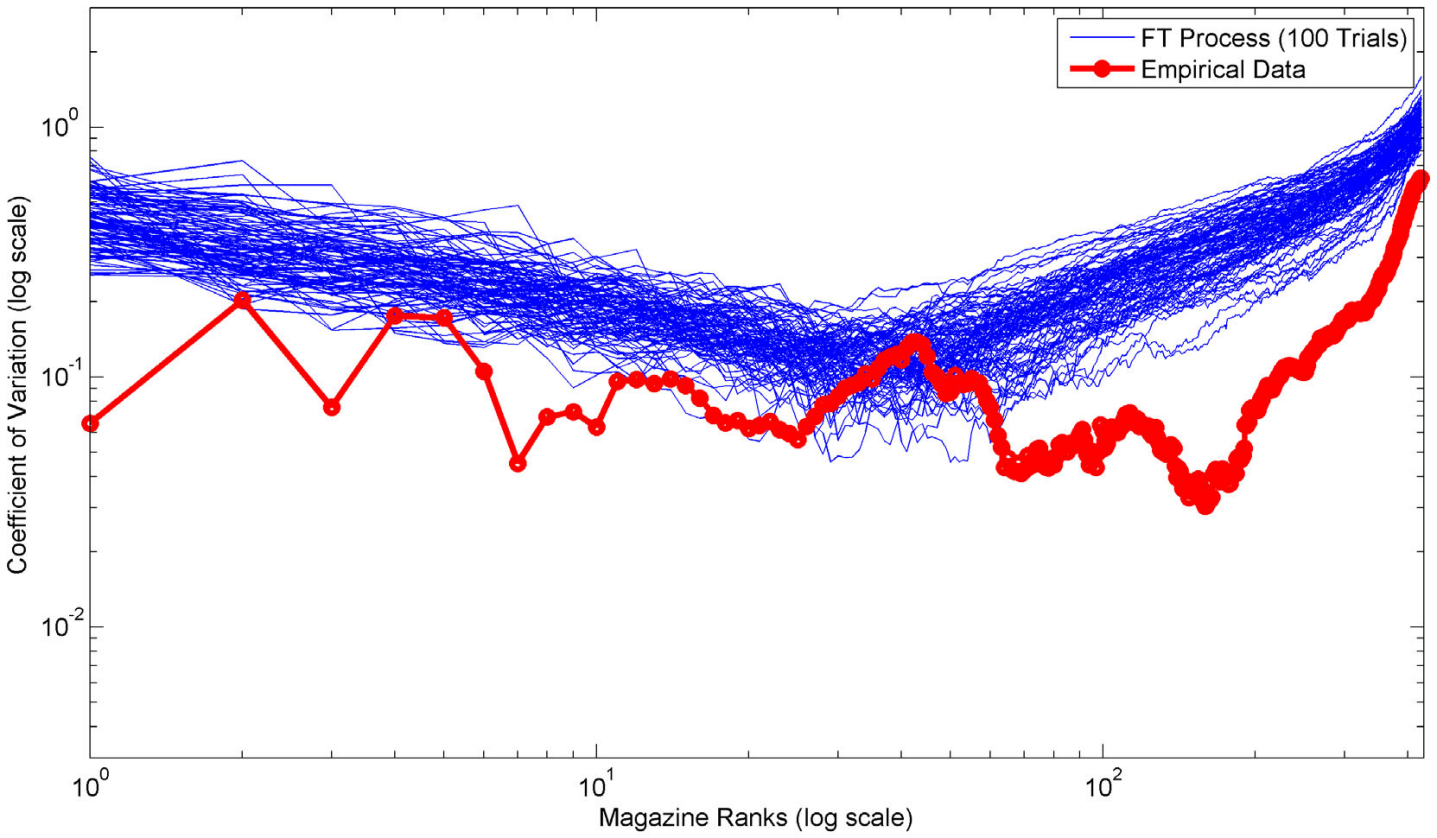
Empirical and simulated coefficients of variation for ranks of 2000–2012 US magazine circulation revenue estimates. Plot of the coefficients of variation of ranks for the 2000–2012 US magazine circulation revenue dataset. The blue lines are simulated coefficients for 100 FT process trials, while the red line is the coefficients of the empirical data.

We compare the variation of each empirical dataset with the variation of repeated trials of the fitted FT processes. The variation was measured by the coefficient of variation (CV) at each rank over the entire data period. The coefficient of variation is a common normalized variation (or dispersion) measure defined as the ratio of the standard deviation to the mean. In cases where the number of ranks varied during different years, the CV was calculated only up to maximum common rank of all observations. Figures 6,7, and 8 show that the empirical variation and FT process variation follow a roughly similar pattern through most ranks.

In both the empirical distributions and FT process, the very high variation of the initial ranks is due to large outliers which, by definition, should appear at the head of rank distributions. Often these outliers are the combined result of several rare conditions or factors. For example, the movie Avatar earned significantly more than any other movie released between 2002 and 2012 due to a combination of excellent release timing, weak competition, 3D technology hype, higher 3D tickets prices, and a world famous director [23].

**Figure 9:**
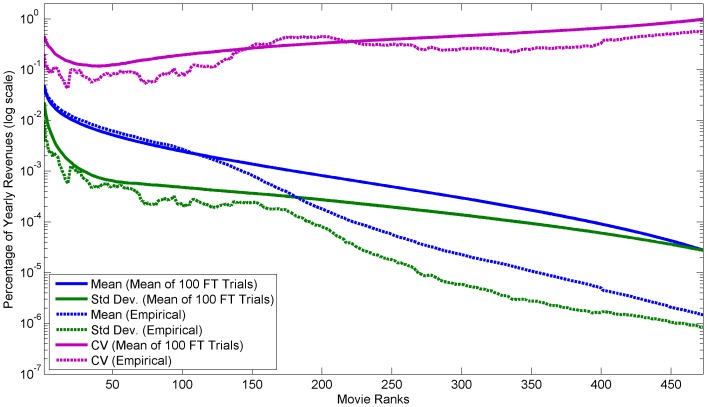
Empirical and simulated average, std. dev., and coefficient of variance for ranks of 2002–2012 US theatrical earnings. Plot of the Empirical and simulated average, std. dev., and coefficient of variance for ranks of the US theatrical earnings dataset. The solid lines are the average of 100 FT process trails, while the dashed lines are of the empirical data.

In contrast, in empirical distributions small outliers at the bottom ranks are generally limited by the data collection methodology or limits of the real-world process. Whereas, in the FT process small outliers are limited by the underlying gamma distribution skewness and the minimum interval size constraint. Furthermore, the slow increase in variation throughout the middle ranks is due to the differences in the mean and standard deviation slopes. Figure 9 depicts the mean and standard deviation of the US theatrical earnings dataset over all ranks.

In general, the variations of the empirical datasets are lower than the average FT process variations. Table 2 compares the total variations from the empirical datasets and the FT processes. The lower variations are likely due to short and long range dependencies. In other words, the longitudinal observations are not truly independent. These dependencies are especially important in interpreting the empirical variation and understanding the underlying real-world mechanistic processes.

**Table 2:** Total empirical and FT process variation for longitudinal datasets.

Dataset	Empirical Variation	FT Process Variation^2^
French Book Sales^1^	3.07	4.59±1.23
US Theatrical Earnings	128.17	197.28±34.39
US Magazine Circulation Revenue Estimates	65.11	191.40±29.25

1 Includes only the variation of the 17 ranks with available empirical data; ^2^Average and Standard Deviation of 100 Trials.

Broadly, we can categorize these empirical dependencies into either direct or indirect dependencies. A direct dependency implies that the same rank value spans several longitudinal observations. A notable example is the first rank of the US magazine circulation revenue dataset, which Figure 8 shows has very low variation over time. In fact, the same magazine (People) held the first rank for seven consecutive years (2005–2012) [20]. Furthermore, the circulation revenue of magazines should inherently be more stable due to their subscription based business model. Importantly, data collection procedures can sometimes determine whether direct dependencies are actually present. For example, the US theatrical earnings dataset does not contain direct dependencies because the gross earnings for each movie were always fully counted toward the year of the initial release date. In contrast, an indirect dependency implies that certain characteristics of the real-world mechanistic process remain fundamentally the same. For example, the distribution mechanisms and sales point strategies of large French book publishers could remain constant for several years thus contributing to longitudinally similar distributions.

In any case, the magnitude of the empirical variation will generally be smaller than the FT process average variation and this should be taken into account when utilizing the FT process.

### Process Limitations

A significant limitation of the FT process is that the process can only create rank-frequency distributions with reciprocal head and tail slopes (ignoring the minimum size limit). This limitation is inherent in the FT process itself due to the process’s common fracturing point distribution. This limitation could be addressed by utilizing different fracturing point distributions for different fractures depending on the magnitude of the interval. However, this addition would add significant complexity to the process, thus potentially undermining the processes strengths of simplicity and understandability. We leave this exploration for future work.

### The Empirical Processes in Reality

An important question is whether the FT process roughly mirrors the real-world processes of the studied datasets or whether the process merely happens to produce similar rank-frequency distributions.

Historically, researchers have described many different processes that produce particular rank-frequency distributions. Ref. [2], for example, details a wide range of processes that produce power law rank-frequency distributions. A prominent example is the Yule process which uses a form of preferential attachment and has been utilized to explain the distributions of city sizes and article citation counts [2]. In other words, the appearance of a power law rank-frequency distribution, for example, generally does not imply a particular type of underlying process or even any significant mechanistic complexity in the underlying process [24]. Instead, the specific data source and context must be thoroughly examined.

In the case of US theatrical earnings, each process cascade could be interpreted as an individual (supply side) business decision or event that determines the combination of resources (financial, human, etc.) devoted to each movie. For example, the initial cascade could represent the division of gross movie industry resources into movie studio alliances. A similar argument can be made for the French book sales data and music popularity data and several other datasets.

Realistically though, many of these behavioral based databases might be more accurately represented by a (demand side) detailed preferential attachment process. Such processes have been used utilized extensively to model human social networks [25] [26]. However, the strength of the FT process is that the process can model many rank-frequency distributions well enough while still being simple and easy to understand. In other words, empirical datasets don’t necessarily need underlying mechanistic fragmentation processes for the FT process to be utilized.

### Economic Simulation

**Figure 10:**
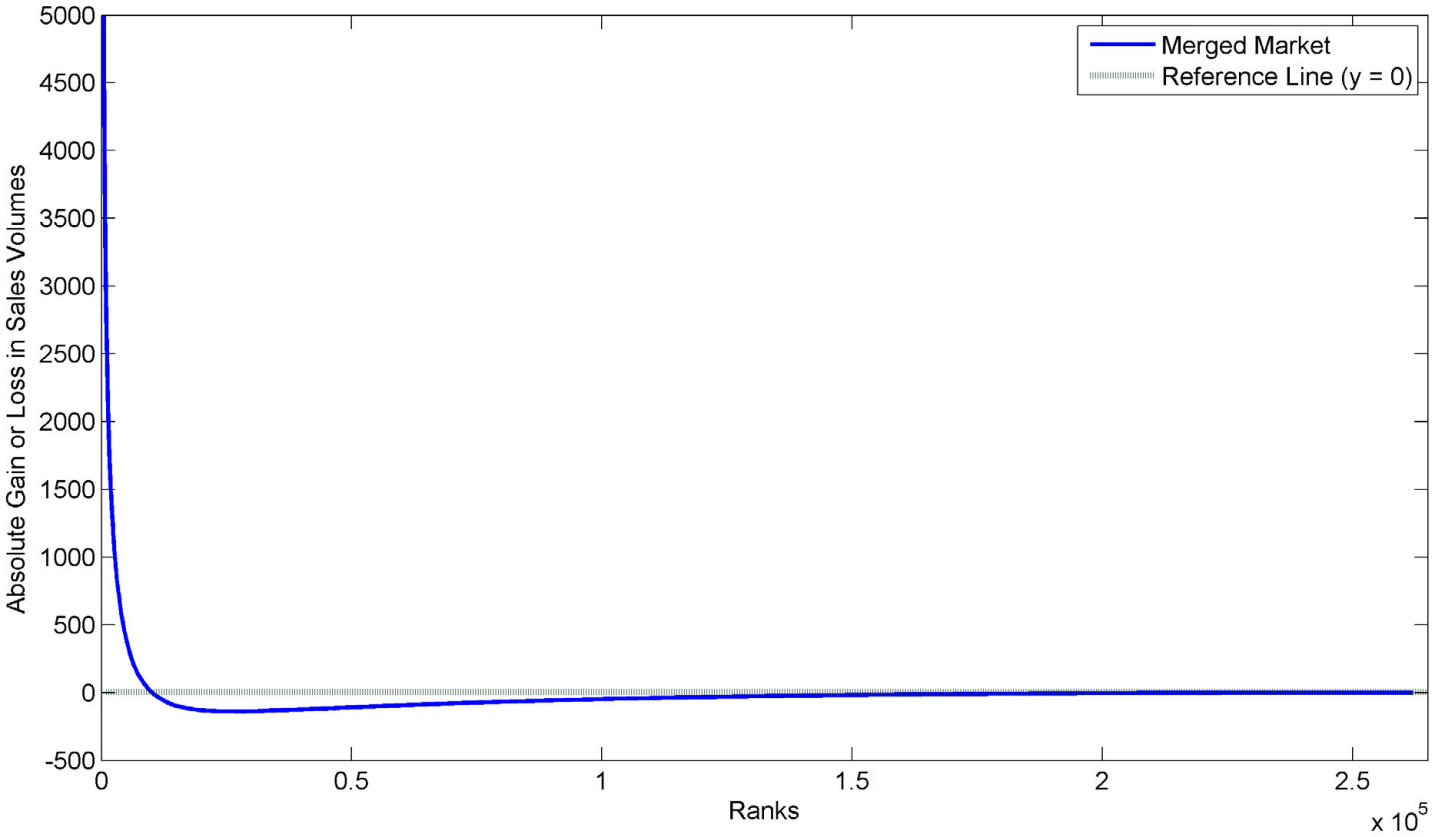
Average absolute gains or losses for each rank in the single merged market. Plot of the absolute gains or losses of a single merged market compared to four smaller markets. Each small market being identical and based on the 2003 French book sales dataset.

The definition of the FT process ranks as a set of goods in a single marketplace, as in the French book market, allows for experiments in market dynamics. For example, we simulated the merger of four identical French book markets, with each market based on the 2003 French book dataset, into a single large market. In practical terms, we compared the combined and resorted ranks of four FT process simulations against a single larger FT process simulation. Figure 10 depicts the average (over 50 trials) absolute gains or losses for each rank in the merged market. Clearly, the biggest winners of such a market integration are the very top ranks. In fact, in this example, all books below the top 10% of ranks sustain absolute sales volume losses (if we assume the total market volume remains the same).

These results align with theories that postulate that market integration will increase the so called “superstar” effect. From an FT process viewpoint, this integration implies a larger overall market and several additional fracturing steps. If the fracturing process remains unchanged, increasing inequality is an inevitable consequence.

Often in practice, the full set of empirical ranks is not available due to, for example, privacy concerns or business confidentiality reasons. In cases where only the head ranks are available the decision over which model or process to utilize becomes much more difficult. In economic situations, the underlying supply and demand side effects can give hints as to the tail behavior. For instance, the French book sales and US theatrical earnings datasets both have significantly truncated tails. This tail behavior is potentially related to physical distribution bottlenecks and high marginal inventory costs. (Online French book sales accounted for only 4% of total French book sales in 2007 [15] and thus marginal inventory costs for the dominant brick-and-mortar stores were still very important.) In contrast, the Audioscrobbler and YouTube datasets, both digital services with few distribution bottlenecks and low marginal inventory costs, have non-truncated tails. The FT process is typically a better fit for datasets with significantly curved or truncated tails.

## Discussion

Overall, the FT process provides a simple yet useful model for many empirical rank-frequency relationships; especially for datasets with concave rank-frequency distributions (on a log-log scale) and reciprocal head and tail slopes. Furthermore, the FT process can simulate the longitudinal variation of empirical datasets since the process’s longitudinal variation roughly follows the same shape as many empirical datasets variations.

In terms of further practical applications, rank-frequency models are increasingly being applied in empirical economics research [27] [28] [15]. The expansion can be partly explained by the popularization of long tail business models, which often utilize rank-frequency demand (or popularity) curves. In a long tail business model, a business typically sells less of each individual product but sells a much larger variety of products, thus the description of the long tail as selling less of more, the online music business is a salient example [29]. In these cases, knowledge of the longitudinal variation of ranks in these demand curves is particularly interesting and useful.

For instance, predicting the magnitude of the first few rank elements is typically difficult due to high variation. As mentioned, by definition, the outliers of the underlying size-frequency distribution will be placed at the ends of the related rank-frequency distribution. However, an accurate prediction of these first ranks is useful in, for example, quantitative financial risk analysis and supply/capacity management. Imagine a situation with only two previous longitudinal observations, an obvious method to predict future ranks is to average the previous two observations.

The confidence placed in these predictions should be based on the expected variation of these ranks. In the absence of variation information from a similar business sector, a general variation estimate based on the FT process could be utilized as a starting point. We leave these additional examples and methods for future work.

With regard to overall understandability, the FT process has real-world analogues such as the repeated fracturing of rocks into sediment that are easily observed and understood. Furthermore, the fitting parameter can be understood as an analogue to the physical distribution of force causing the fracturing. In other words, the fracture will probably (though not certainly) occur where there is the greatest application of force over area (pressure).

Finally, in terms of transparency, a formal and full mathematical treatment of the FT process is still needed.

## Methods

### FT Process Description

The FT process starts with a single unit interval which is then fractured into two intervals with size 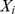 and 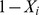. Where 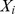 is the transformation of an independent standard uniform random variable by a piecewise linear function. This piecewise linear function is defined as
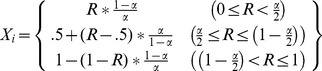



where 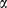 is the fitting parameter on 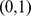 and *R* is the standard uniform random variable. Each of these two intervals is then similarly fractured into two smaller intervals to create four intervals. This recursion continues until the final time step after which there are 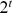 total intervals (where 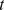 is the total number of steps with the initial interval being step 0). If the number of empirical ranks is not a multiple of 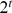 then the final fracturing occurs up to the number of empirical ranks. In other words the number of process intervals is adjusted to match the number of empirical ranks. These intervals are then normalized by the sum of the empirical distribution and finally sorted by size to produce a rank-frequency distribution.

**Figure 11:**
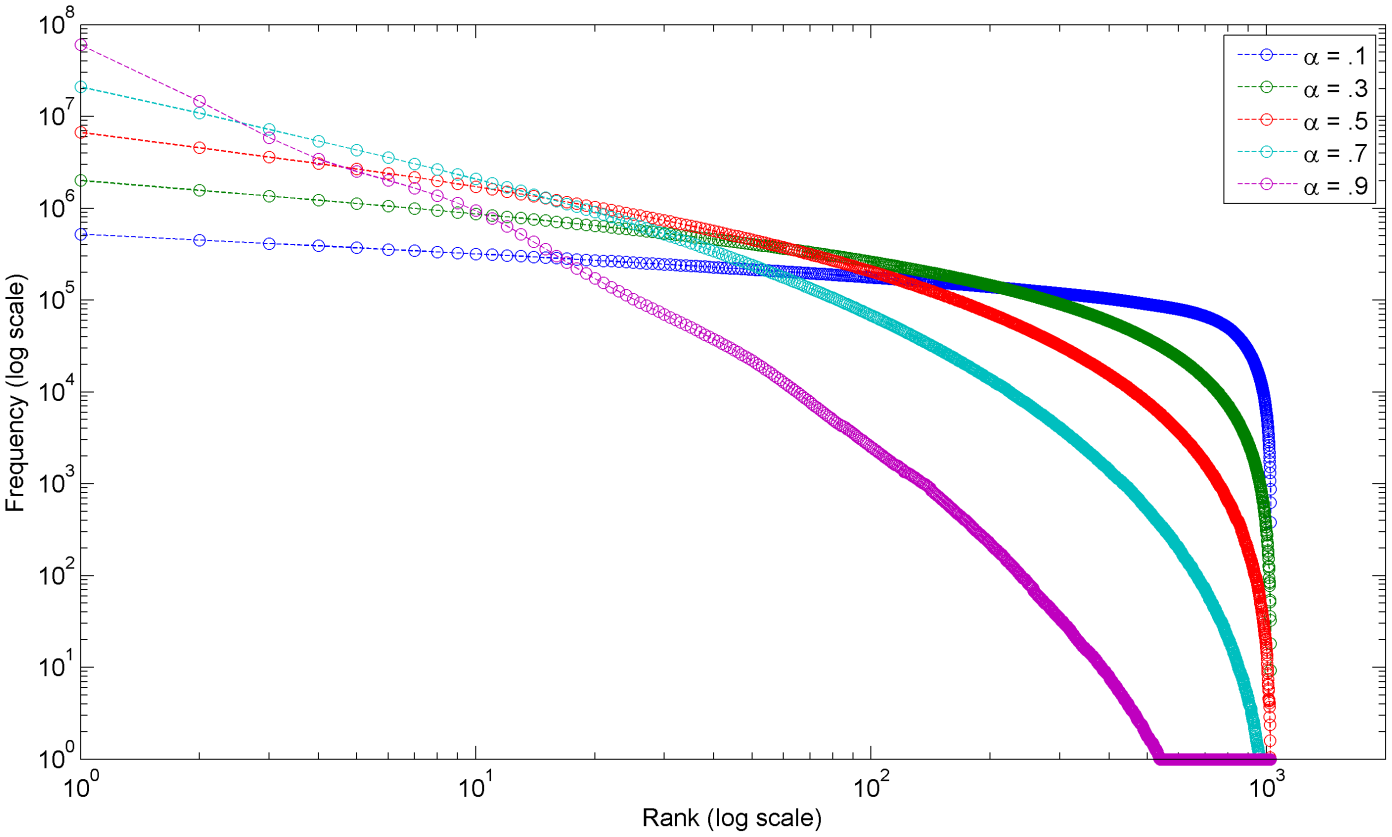
Rank-frequency distributions from FT process with varying fitting parameter α. Plot of several rank-frequency distributions from FT process with varying fitting parameter α but a constant number of intervals. Each distribution is the average of 1000 FT process simulations.

The shape of the resultant rank-frequency distribution is determined by the fitting parameter, 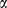, of the transform. This parameter is varied for each dataset to fit the rank-frequency distribution to the empirical rank-frequency distribution. The effect of a large 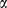 is relatively more fracturing near the middle of intervals and thus less variation between the interval sizes; while the effect of a small 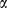 is more fracturing near the extremes of intervals and thus more variation in the interval sizes. Interestingly, if 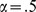 then the resulting distribution simplifies to the original standard uniform probability distribution (since the transformation function becomes unity). Figure 11 shows rank-frequency distributions with varying fitting parameter, 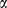, values but a constant number of intervals. Table 3 shows the fitting parameter values utilized for simulating the empirical data and we have found that these are typical values.

**Table 3:** FT process, power law, and DGBD parameters for the fitted datasets.

	FT Process	Power Law		DGBD		
Dataset	α	ln A_i_	βi	ln A_i_	βi	γi
French Book Sales (2003)	.50	13.68	−.5813	−36.23	−.5728	−4.500
US Theatrical Earnings (2002)	.52	20.94	−1.147	1.581	−.6256	−3.024
Census Respondents Last Name Frequency	.48	15.54	−.8246	6.067	−.7808	−.7816
Audioscrobbler Music Artist Play Frequency	.58	16.70	−1.026	−6.185	−.9619	−1.581
UMASS Amherst YouTube Video Request Frequency	.25	6.473	−.5216	2.722	−.5046	−.3053
US Magazine Circulation Revenue Estimates (2000)	.35	20.60	−.8662	15.50	−.7428	−.7648

The FT process also imposes a minimum size for the generated intervals. This constraint helps simulate real-world limitations and is specific to the empirical dataset being fit. For example, a bookstore does not typically sell fractions of a single book. In Figure 11, the purple curve with 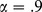 illustrates a minimum size constraint (in this case a minimum size of 1).

In practical computational terms, the FT process naturally lends itself to a recursive implementation. We provide a simple recursive open source licensed implementation in Perl as a supporting information file (Supporting Code S2).

### Fitting Methodology

#### Discrete Generalized Beta Distribution (DGBD)

The discrete generalized beta distribution represents a useful comparison and reference point. Ref. [30] details the original development of the distribution and some applications. The beta distributions fitting parameters were estimated through a multiple linear regression method described in [21]. In essence, the method estimated the parameters 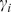, 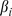, and 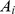 through linear regression of the natural log transformation of the discrete generalized beta distribution. This transformation is
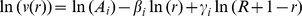



where 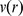 is the value of rank 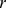, 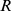 is the maximum rank, and 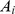 is a normalization constant. As mentioned, regression weights were also used to emphasize the head ranks. The weights were reciprocals of the ranks, in other words for ranks 1, 2, 3, 4 the weights were 1, 1/2, 1/3, 1/4 and so forth.

#### Power law model

The power law model represents a classic one parameter model. The parameter fitting was performed through a similar multiple linear regression method as the DGBD fitting and the natural log transformation is
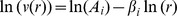



where 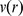 is the value of rank 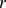, 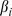 is a shape parameter, and 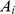 is a normalization constant. The utilized weighting scheme was also the same. As evident from the previous equations, the power law model represents a special case of the DGBD model where 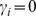.

#### Weighted coefficient of determination (*R* ^2^)

The weighted coefficient of determination (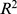) is
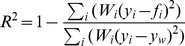


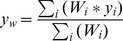



where 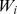 is the specified weight, 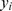 is the empirical value, 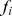 is the predicted value, and 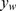 is the weighted mean. This formulation is only one of many possible definitions, [21] provides an expanded explanation of many formulations.

### Empirical Data

The utilized empirical datasets are described in detail including the exact dataset source and dataset limitations. Table 4 summarizes the basics of each dataset.

**Table 4:** Summary of all empirical datasets.

Dataset	Rank Unit	Value Unit	Range	Source
French Book Sales	Book	Sales Volume (books)	2003−2007	[15]
US Theatrical Earnings	Movie	Gross Earnings (USD)	2002−2012	[16]
Census Respondents Last Name Frequency	Last Name	Census Respondents	2000	[17]
Audioscrobbler Music Artist Play Frequency	Artist	Plays	2005^1^	[18]
UMASS Amherst YouTube Video Request Frequency	Video	Requests	2007−2008^2^	[19]
US Magazine Circulation Revenue Estimates	Magazine	Circulation Revenue (USD)	2000−2012	[20]

1 The Audioscrobbler dataset includes all information aggregated over a period of several years up to May 2005; ^2^The UMASS Amherst YouTube dataset consists of aggregate YouTube video request frequencies over several observation periods from the fall of 2007 to spring 2008.

The French book sales dataset consists of sales volume data of 16 distinct ranks for the years 2003 to 2007. The dataset was released by [15], which obtained the original data from the French subsidiary of market research company GfK. The dataset covers only sales of physical books (from brick-and-mortar or online stores); however, in 2007 digital book (often called eBook) sales in France were negligible.

The US theatrical earnings dataset consists of gross domestic box office earnings data for US movies for the years from 2002 to 2012. The dataset was released by Box Office Mojo [16], a subsidiary of Amazon, which systematically tracks box office earnings data. Box Office Mojo provides domestic box office earning data is several different formats and we use the total domestic grosses viewed by release date. Importantly, in this format the gross earnings for each movie were fully counted toward the initial release year [31]. Furthermore, the dataset covers only theatrical earnings (in other words earnings from movie theatre ticket sales) and thus excludes earnings from subsequent home movie rentals or sales.

The census respondents last name frequency dataset consists of the frequency of almost all last names of census respondents of the 2000 US census. The dataset was released by the US census bureau [17]. For confidentiality reasons, the census bureau did not include last name information for those names with frequency of less than 100.

The Audioscrobbler artist play frequency dataset consists of the frequency of plays for artists through the Audioscrobbler music recommendation plugin over several years up to May 2005. The dataset was released by Audioscrobbler in May 2005 [18]. At that time the plugin had over 150,000 users. Unfortunately, Audioscrobbler was acquired by LastFM in 2005 and no additional datasets have been released.

The UMASS Amherst YouTube dataset consists of the aggregate frequency of client requests for YouTube videos originating from the University of Massachusetts Amherst campus network during several dispersed observation periods during fall 2007 and spring 2008. The dataset was released by researchers into the public UMASS Trace repository [19]. Ref. [32] performed the initial collection and analysis of the dataset.

The US magazine circulation revenue estimates dataset consists of circulation revenue estimates for most US magazines over the years from 2000 to 2012. The dataset was released by The Association of Magazine Media, a nonprofit industry association [20].

### Analytical Form of FT Process

Finally, we attempt to derive an analytical form for the FT process rank function. However, we find that a simple analytical approximation is unlikely to exist due to the complexity added by the piecewise transformation function. Though we are able to derive an analytical approximation for a special case of the FT process.

### Background

In order to derive the rank function we need to derive the probability distribution function (hereafter PDF) of interval sizes from a single FT process trial (since the rank function is a simple transform of the corresponding cumulative distribution function (hereafter CDF)). Unfortunately, the resultant interval sizes from a single FT process trial are by definition interdependent. This interdependence results in a non-standard distribution (often with altered variance, see Theorem 2 in [9] for an example from a related fragmentation process) that can be difficult to express due to the necessity to utilize recurrence relations. However, we can approximate the required PDF by the interval size PDF of a single interval over many independent FT process trials. In essence, this approximation assumes interval independence. Naturally, the accuracy of this approximation depends on the number of cascades (and thus number of intervals) as we discuss later.

### PDF of a Single Interval over Independent FT Process Trials

Thus we first attempt to derive this interval size PDF approximation for an FT process with 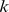 cascades (or steps).

Recall that during each cascade we fracture each interval by multiplying the interval by 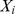 and 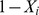, where 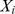 is the transformation of a standard uniform random variable by the piecewise linear function
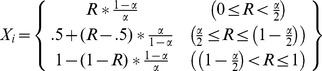



where 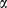 is a fitting parameter and 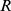 is the standard uniform random variable. Thus our first step is deriving the probability distribution function of this transformed random variable 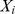. We can utilize the general univariate change of variable method in a piecewise approach (due to non-differentiable corners between the subfunctions of the transformation) [33]. The generalized change of variable method formulation is
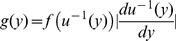



where 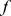 is the PDF of the original (in our case standard uniform) random variable, 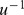 is the inverse of the transformation function, and 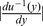 is the absolute value of the Jacobian of the inverse of the transformation function. The PDF of 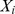 is thusly
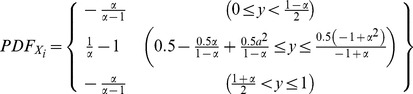



Where 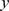 is utilized due to the inverse transformation function. Next we derive the probability distribution function of the product of two independent random variables each defined by 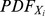, in other words the PDF of 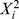 (thus describing an interval after two cascades).

Deriving the PDF of the product of random variables is often laborious because the resulting distribution is typically piecewise defined. Thus we utilize the symbolic statistical package MathStatica (built on top of the mathematical platform Mathematica) [34]. The MathStatica package symbolically utilizes Rohatgi’s famous expression for the product of two continuous independent random variables
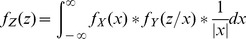



where 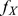 is the PDF of one random variable, 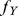 is the PDF of the other random variable [34] [35]. The resulting PDF of 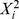 is an 11 subfunction piecewise defined function. We detail the function in Supporting Text S1 due to space limitations. We then attempt to derive the PDF of 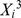 (thus representing an interval after three cascades), again using MathStatica, however, we find that the calculation is already computationally intractable on a standard desktop computer. The intractability is likely due to the increasing complexity and large number of subfunctions and subdomains. Thus a simple analytical form for the approximation PDF of interval size with 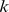 cascades is unlikely to exist or to be of practical use, and we are not able to derive an analytical form approximation of the FT process rank function. However, we can further examine the special case of the FT process where 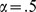. In this case the linear transformation reduces to unity and the original standard uniform random variable is recovered. The product of 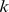 independent standard uniform random variables is a well-studied problem with a closed form [36]

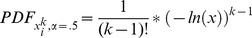



Interestingly, this form shows that, in this special case, the natural logarithm of the size of an interval (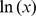, hereafter the LN-size) follows a Gamma distribution.

### Approximation Accuracy

We can also still examine the accuracy of this special case, 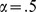, PDF approximation.

Notice that if all the resultant intervals from a single process trial were independent then each interval LN-size would simply be a random variate from independent and identical gamma distributions and thus also follow the above gamma distribution (with a sample size of 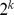 rather than the number of FT process trials).

**Figure 12:**
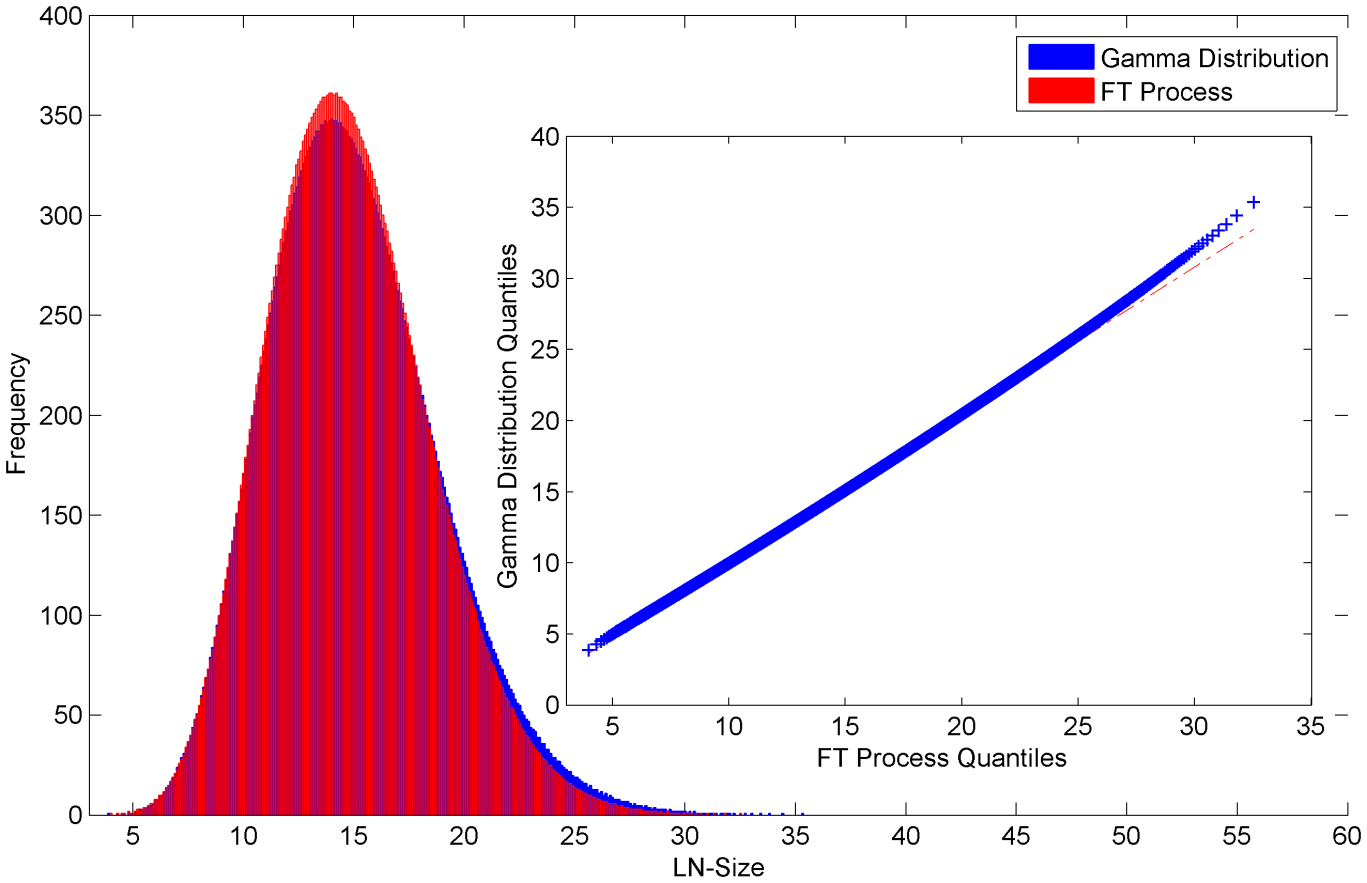
LN-size of the Gamma Distribution and FT Process. Histogram and QQ-plot (inset) of the LN-size of the Gamma Distribution (average of 10000 sorted distributions) and FT Process (average of 10000 sorted trials).

However, as mentioned, the interval sizes from a single process trial are by definition interdependent and the LN-size distribution from a single process trial deviates from the above gamma distribution. Illustratively, Figure 12 shows the average of 10000 FT process trials (each trial result was sorted before summing) compared with the average of 10000 gamma distributions (again each distribution was sorted before summing). The sorting before the summing of each trial or distribution basically means the empirical CDFs (empirical distribution functions) are averaged. The average of empirical i.i.d. CDFs almost surely converges pointwise to the true CDF by the strong law of large numbers [37]. The corresponding PDFs are shown in Figure 12. As expected, the distribution of a single process trial shows less dispersion than the gamma distribution due to the interdependencies between the resultant intervals. Numerically, we have found that the relative difference between the std. dev. of the single process trial and the gamma distribution depends on the number of steps 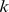. And as expected, the difference is larger for smaller values of 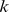.

### Rank Function Approximation

Finally, we can also utilize this special case, 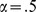, PDF approximation to create an approximation of the process rank function for 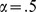. First we integrate the PDF with respect to a third variable 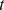 over the limits from 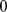 to 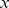 obtain the CDF as
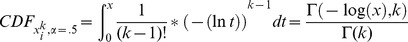



Where 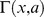 is the upper incomplete gamma function (hereafter UIGF) defined as
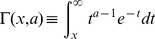



And 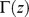 is the complete gamma function defined as
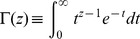



Notice that the UIGF could also be replaced by the lower incomplete gamma function through the use of, for example, a normalized gamma function identity (8.2.5 in [38]). The lower incomplete gamma function is more commonly seen in formulations of gamma CDFs. Next, according to [21], the rank function can be derived from the CDF by solving
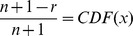



for 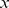 as a function of 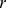. Where 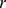 is the rank and 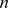 is the total number of ranks (in our case 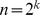). Thus we must solve
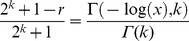



for 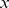 as a function of 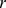. First notice that the right hand side of the equation is a form known as the normalized UIGF (since the UIGF is essentially normalized by the complete gamma function). Many common mathematical packages such as MATLAB and Mathematica have functions that inverse this normalized UIGF [39] [40]. However, unfortunately no simple analytic form exists for this inverse function and these mathematical packages only find numerical approximations. MATLAB’s approximation function, for example, uses Newton’s method [40]. In any case, we inverse the normalized UIGF on the second parameter (notice importantly that this parameter is the integration limit parameter of the UIGF and not the integrand parameter of the UIGF) to give
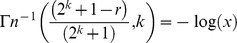



Finally trivial operations then give the final approximation rank function form of
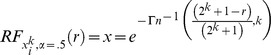



## Supporting Information

Text S1 Probability Density Function of FT Process after Two Cascades.
(DOC)

Code S1 Recursive Implementation of FT Process in Perl.
(PL) Click here for additional data file.
