# Supplementary material for: Exploring Empirical Rank-Frequency Distributions Longitudinally through a Simple Stochastic Process
Source: PLoS One. 2014 Apr 22;9(4):e94920. doi: 10.1371/journal.pone.0094920 (PMC3995693; doi:10.1371/journal.pone.0094920)
Supplement: Text S1 — Probability Density Function of FT Process after Two Cascades. (DOC) [file pone.0094920.s001.doc]

Note that some subfunctions and subdomains are present in both parts of the function, there are 11 unique subfunctions.

For $3-\sqrt{2}<\alpha<1$

For $0<\alpha<3-\sqrt{2}$

$$PDF_{x_{i}^{2}}=\left\{\begin{matrix} \frac{a^{2}Log\left[\frac{2}{a+1}\right]}{2y} & \left(y=\frac{1}{4}\left(1-2a+a^{2}\right)\right) \\ \frac{2(1 -a)^{2}Log\left[-\frac{2}{a-1}-1\right]+a^{2}\left(2Log\left[\frac{2}{a+1}\right]-Log\left[\frac{4y}{(a-1)^{2}}\right]\right)}{(a-1.)^{2}} & \left(0<y<\frac{1}{4}\left(1-2a+a^{2}\right)\right) \\ \frac{2a^{2}(1 -a)^{2}Log\left[\frac{0.25 -0.25a^{2}}{y}\right]+(1 -a)^{4}Log\left[\frac{y}{(1-a)^{2}}\right]+2a^{4}Log\left[\frac{2}{a+1}\right]+(a(a(Log[4]a-Log[256])+Log[4096])-Log[256])a+Log[4]}{(a-1.)^{2}a^{2}} & \left(\frac{1}{4}\left(1-2a+a^{2}\right)<y<\frac{1}{4}\left(1-a^{2}\right)\right) \\ -\frac{a^{2}Log\left[y\right]}{\left(-1+a\right)^{2}} & \left(\frac{\left(1+a\right)}{2}<y<1\right) \\ \frac{a^{2}Log\left[\frac{4y}{\left(1+a\right)^{2}}\right]}{4y^{2}} & \left(y=\frac{1}{2}-\frac{a}{2}\right) \\ -\frac{2a^{2}Log\left[-\frac{2y}{-1+a}\right]}{\left(-1+a\right)^{2}} & \left(y=\frac{1}{4}\left(1+2a+a^{2}\right)\right) \\ \frac{a^{2}(2-4a)Log[y]+2a^{2}(1-a)^{2}Log\left[\frac{1}{1.-a^{2}}\right]+(1-a)^{4}Log\left[\frac{(a+1)^{2}}{y}\right]-2a^{4}Log\left[-\frac{1}{a-1}\right]+\left(\text{-}Log\text{[256]a+}Log\text{[256]}\right)a-Log[4]}{(a-1.)^{2}a^{2}} & \left(\frac{1}{4}\left(1-a^{2}\right)<y<\frac{1-a}{2}\right) \\ \frac{\left(2-4.a\right)Log\left[\frac{1}{1-a}\right]+\left(2-4a\right)Log\left[1+a\right]-a^{2}Log\left[y\right]}{\left(-1.+a\right)^{2}} & \left(\frac{\left(1 +2a+a^{2}\right)}{4}<y<\frac{\left(1-a\right)}{2}\right) \\ \frac{-Log[4]+Log[16]a+\left(1+\left(-2-a\right)a\right)Log\left[1+a\right]+\left(-1+2a\right)Log\left[y\right]-\left(1-a\right)^{2}Log\left[\frac{y}{1+a}\right]}{\left(-1+a\right)^{2}} & \left(\frac{1-a}{2}<y<\frac{1+a}{2}\right) \end{matrix}\right\}$$
